# Medical grade footwear: does it prevent ulcer recurrence? An Australian perspective

**DOI:** 10.1186/1757-1146-4-S1-P11

**Published:** 2011-05-20

**Authors:** Melissa Coyle, Julia Firth

**Affiliations:** 1 St Vincent’s, Podiatry Department, PO Box 2900, Fitzroy, Victoria, 3065, Australia

## 

Medical grade footwear is frequently used in the prevention and treatment of ulceration in the high risk foot. Many factors can contribute to the breakdown of tissue in the foot which leads to ulceration. Foot deformity, reduced joint mobility, mechanical stress and strain, peripheral neuropathy, vascular disease and increased pressure are common factors. The right footwear can aid in reducing the effects of these factors and assist in prevention of tissue breakdown. The St Vincent’s (Melbourne) Podiatry Department introduced an in house footwear range 18 months ago. This study is to determine the effect this range of extra depth and width footwear has had on patient’s foot health. The St Vincent’s Podiatry Department is conducting a retrospective study on 40 high risk patients over a 12 month period in an acute hospital setting. All patients have been prescribed medical grade footwear. Patients who were determined high risk are assigned a risk classification 0 (low risk) – 3 (high risk). The aim of this study was to identify those patients who had a poor outcome such as further ulceration or amputation since being issued with medical grade footwear within this time frame. From this retrospective study we hope to be able to conclude that therapeutic footwear can assist in prevention of foot ulcers if fitted correctly.

